# Reversible Vagal Nerve Stimulation-Induced Vocal Cord Paralysis and Intractable Neck Pain Following a Syncopal Fall: A Case Report

**DOI:** 10.7759/cureus.51489

**Published:** 2024-01-01

**Authors:** Ethan J Houskamp, James M Mossner, S. Katie Bandt

**Affiliations:** 1 Department of Neurological Surgery, Northwestern University Feinberg School of Medicine, Chicago, USA

**Keywords:** case report, pain, vagus nerve stimulation, epilepsy, vocal cord paralysis

## Abstract

Vagal nerve stimulation (VNS) is a well-tolerated procedure for patients with medication-resistant and non-focal epilepsy. It does, however, have potential complications (e.g., hoarseness and cough) thought to be from vagus nerve irritation. These arise postoperatively and generally improve without intervention. If these symptoms present later or do not improve, it suggests a more insidious etiology. Herein we report the case of a patient in their 50s with medication-resistant epilepsy, who subsequently underwent VNS electrode array and pulse generator implantation to aid seizure management. Three years after the initial implantation, the patient experienced vocal cord paralysis and neck pain following a syncopal fall. The pain radiated to their jaw and chest and was eliminated when their VNS was turned off. The patient was taken to the OR for removal and replacement of their entire VNS system. Their original electrodes were unable to be removed secondary to being scarred in place. The patient’s preoperative pain symptoms completely resolved after the removal of their old VNS and implantable pulse generator (IPG) and replacement with a new system 14 days postoperatively. While short-term postoperative sequelae and lead fractures/displacements have been reported in the literature, this is the first case to our knowledge of a patient experiencing a likely symptomatic traction injury without displacement of the VNS coils or obvious vagus nerve injury. Furthermore, the removal and replacement of the entire VNS system led to complete relief of their presenting symptoms.

## Introduction

Up to 30% of patients with epilepsy have drug-resistant epilepsy [1]. In this cohort, patients with multiple, poorly localizable, or generalized epileptogenic foci can be considered for vagus nerve stimulation (VNS). Though the mechanism of action of VNS remains largely unknown, results have consistently demonstrated its effectiveness in reducing seizure burden by more than 50% in up to 63% of patients [2-5].

VNS is a well-tolerated procedure with few short- and long-term side effects [6]. Hoarseness and coughing are the two most frequently cited immediate side effects. Hoarseness is more common in adults and is hypothesized to be from left vocal cord paralysis secondary to reversible vagus nerve dysfunction [7, 8]. Hoarseness that develops later because of trauma suggests potentially irreversible damage to the nerve. A select few case reports have reported on causing hardware failure resulting from trauma [9-11]. Here, we report an unusual case of an adult who experienced a syncopal fall secondary to orthostatic hypotension, causing a suspected traction injury to the vagus nerve leading to VNS malfunction, left vocal cord paralysis, and severe pain radiating from the patient’s neck to their chest.

## Case presentation

History and physical

The patient is an adult in their 50s with a history of coronary artery disease (CAD), myocardial infarction status post right coronary artery stent, hyperlipidemia, and medication-resistant epilepsy, which led to the decision to undergo VNS electrode array and implantable pulse generator (IPG) implantation. The patient was originally treated with brivaracetam, lorazepam, and lacosamide with poor seizure control. They were ultimately deemed a candidate for VNS as a palliative procedure to improve their seizure control given their cardiopulmonary co-morbidities and requirement for lifelong continuous anti-platelet therapy. Their original VNS lead and IPG implant procedure was completed early in 2019 without complications. While seizures persisted, the patient reported their seizure burden as greatly improved after turning on stimulation.

**Figure 1:**
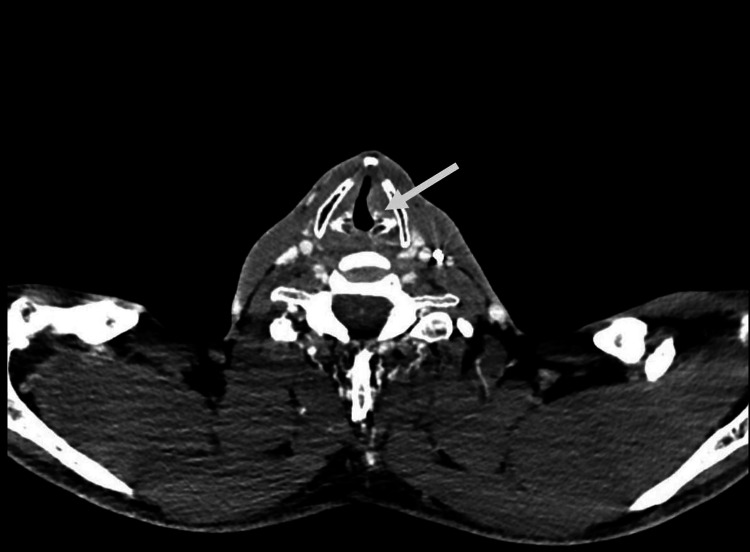
Patient's neck CT Medialization/slight rotation of the true left vocal cord (arrow sign) consistent with left vocal cord paralysis.

Three years after the VNS placement, the patient experienced a syncopal fall in December 2022 secondary to orthostatic hypotension. In February 2023, the patient was referred to the ENT department after developing a lump and choking sensations in their throat. A modified barium swallow study revealed dysphagia, and laryngoscopy identified left vocal fold paralysis. Their symptoms improved but did not ultimately resolve with speech therapy. In March 2023, they began experiencing severe left neck pain with radiation to their left chest, abdomen, and left jaw as well as worsening seizure control. The midline pain came in short intervals and was associated with shortness of breath. Workups by otolaryngology and cardiology identified only vocal cord paralysis. In July 2023, the patient’s VNS output settings were lowered to 1.75 mA from 2 mA for the normal and AutoStim modes and to 2 mA from 2.25 mA in magnet mode with no subsequent reductions in pain. CT imaging completed in August 2023 was concerning for vocal fold paralysis (Figure 1). While the mechanism was not clear, the patient’s symptoms suggested a possible contribution from their VNS device. No evidence of device dislodgement was identified on imaging. Their treating physician team presumed a component of traction injury to the vagus nerve at the time of their syncopal fall due to the temporal association between their symptom onset and this event. As a trial, their VNS was turned off and their symptoms resolved within minutes. After discussing the goals and complications of revision surgery, the patient agreed to have the VNS and IPG replaced.

Surgical procedure and postoperative follow-up

The patient was brought to the OR in August 2023 after undergoing a thoroughly informed consent process. Due to concern for the left vocal cord paralysis, video laryngoscopy was successfully used to intubate the patient on the first attempt. Intraoperatively, the previously implanted VNS electrode array was identified and dissected down to the level of the vagus nerve. The electrode contacts were cut from the electrode wire, which was removed and discarded. There was no evidence of dislodgement of the electrode contacts from the vagus nerve. The electrode contacts remained in situ and an additional length of vagus nerve was dissected cranial to the original electrode array where a new electrode array was implanted. The original IPG was approaching the end of service and was therefore replaced simultaneously. The patient tolerated the procedure without immediate postoperative complications. On postoperative day one, the patient endorsed incisional pain but stated that the left neck, chest, and abdominal discomfort had resolved.

Initially, the patient was trialed to higher, default VNS settings, but developed a sharp, pulling, and painful sensation that occurred every five minutes on postoperative day two. On postoperative day three, the patient met their neurologist who reduced the VNS settings with resolution of the pain symptoms. On postoperative day six, the patient was evaluated by an otolaryngologist who completed a laryngeal videostroboscopy, which identified no new deficits. Small changes to the automatic stimulation (AutoStim) output settings were made in subsequent weeks, with no other setting changes being made. At their most recent clinic visit, they reported a complete resolution of the pain symptoms that originally brought them to the clinic. Their vocal cord paresis persists but it is gradually improving with continued speech therapy. Importantly, their patient’s seizure burden is back to their pre-syncopal baseline.

## Discussion

VNS therapy is an effective treatment for patients with medication-resistant epilepsy, reducing seizure burden by more than 50% in more than half of the patients [2-5]. It is also a relatively safe procedure due to the most commonly reported side effects (i.e., hoarseness and cough) being tolerable and transient, occurring postoperatively but resolving soon afterward [12,13]. A lack of improvement or delayed complications after VNS surgery are rare and suggest a more insidious etiology. This report describes a case of delayed onset of VNS malfunction, vocal cord paralysis, and radiating neck pain following a syncopal fall secondary to orthostatic hypotension. We hypothesize that the traction on the vagus nerve caused by the syncopal fall may have dissected the nerve’s outer layers of connective tissue in such a way that resulted in a more potent and therefore painful stimulation. We find this the most likely explanation as the patient’s symptoms align with the older case reports of discontinuous lead insulation or lead breakage [14,15].

Since the improvements to VNS hardware have made hardware failures much less common, we are only aware of a handful of post-VNS implantation vagus nerve injuries due to trauma or traction. One case of a traction-based VNS injury was reported by Clark et al., which was due to VNS implantation in a young patient without adequate accommodations for growth [16]. The two other cases of traumatic vagus nerve injuries that have been reported were due to nerve sections, one partial and one total, from a fall and initial VNS implantation surgery, respectively [10, 11]. However, neither of these mechanisms was thought to be from traction injury. To our knowledge, this is the first case of VNS malfunction implicating traction on the vagus nerve secondary to a syncopal event in the etiology causing radiating neck pain and permanent vocal cord paralysis.

Multiple established surgical techniques to repair VNS hardware currently exist and have low complication rates [17-20]. Future studies will be necessary to identify if any of these reoperation procedures lead to superior postoperative outcomes.

## Conclusions

With the increased use of VNS over the past three decades, surgeons have encountered greater VNS hardware malfunctions and complications requiring reoperations. As complications from these cases arise, surgeons need to be able to identify irregular complications that may not be found or well-published in the literature. Therefore, it is important that surgeons understand and can identify known symptoms of VNS malfunction, even if it does not present within typical timelines or with known mechanisms.
